# Microarray analysis of the molecular mechanisms associated with age and body mass index in human meniscal injury

**DOI:** 10.3892/mmr.2019.10629

**Published:** 2019-08-30

**Authors:** Peiyan Huang, Jun Gu, Junguo Wu, Lei Geng, Yang Hong, Siqun Wang, Minghai Wang

Mol Med Rep 19: 93-102, 2019; DOI: 10.3892/mmr.2018.9685

Subsequently to the publication of the article, the authors have realized that the grant number in their paper in the Funding section was presented incorrectly: ‘2017MH77’ should have been written as ‘2017MH**Z**77’.

Therefore, the funding information in the declarations section should have appeared as follows: ‘The present study was funded by the Minhang District Natural Science Project (grant no. 2017MHZ77)’.

The authors regret that this error was not noticed prior to the publication of their paper, and apologize for any inconvenience caused.

